# Poulticing

**Published:** 1882-09

**Authors:** Otto Arnold


### CORRESPONDENCE.

### POULTICING.

*Editor Register* :

As a reply to "K. C. M.," in the August REGISTER, regarding poulticing, I would say that it has a decided influence upon the tissues with which it is in contact and as the poultice is warm or cold, will draw to or repel pus from the surface.

Hot applications in the form of poultices tend to expand the tissues and blood vessels, thereby favoring stasis in these parts, while cold applications have a contrary effect—contracting the tissues, thereby driving away excess, and preventing engorgement. The case in question might have been successfully controlled by external applications alone—until the offending tooth was accessible—or by the addition of internal treatment and proper regime.

Quinine sulphate, given in antipyretic doses; saline laxatives, hot and stimulating pediluvia, either of which alone or in combination wield an influence that is antiphlogistic. If a more desperate method is called for—anæsthesia—to relax the muscles and afford sufficient room to extract the tooth would be the proper thing to do.

The ignorance of the ordinary physician regarding the application of medical science to dental and oral pathology, is deplorable to say the least; hence, the importance of a knowledge of medicine and surgery in dentistry. OTTO ARNOLD, D.D.S.

